# The Effect of Inquiry-Based Stress Reduction on Teacher Burnout: A Controlled Trial

**DOI:** 10.3390/brainsci10070468

**Published:** 2020-07-21

**Authors:** Lia Schnaider-Levi, Ariel B. Ganz, Keren Zafrani, Zehavit Goldman, Inbal Mitnik, Benjamin Rolnik, Shahar Lev-Ari

**Affiliations:** 1 Department of Health Promotion, School of Public Health, Sackler Faculty of Medicine, Tel-Aviv University, Tel-Aviv 69978, Israel; liamerkaz@gmail.com; 2 Department of Genetics, Stanford University, Stanford, CA 94305, USA; abganz@stanford.edu (A.B.G.); rolnik@stanford.edu (B.R.); 3 Begin High School, John Kennedy Street 8, Rosh Ha’Ayin 4852028, Israel; kerenzafrani25@gmail.com (K.Z.); zehavitgoldman@gmail.com (Z.G.); mitnik.inbal@gmail.com (I.M.)

**Keywords:** burnout, teachers, mindfulness, inquiry, IBSR, the work, stress, well-being

## Abstract

Burnout is a well-known phenomenon with significant social, biological and economic costs. In particular, teacher burnout is associated with unfavorable mental health outcomes and economic costs due to reduced hours and teacher turnover. This study investigated the effect of an Inquiry-Based Stress Reduction (IBSR) cognitive-reframing program on teacher burnout using a quasi-experimental design. Fifty-three teachers participated in a prospective intervention with a passive control group. The intervention group completed a 12-week IBSR program with 4.5 h of weekly engagement. Relative to control, teachers in the intervention group showed greater improvements in emotional exhaustion (18.8 ± 5.2 to 15.9 ± 5.7 vs. 16.0 ± 4.8 to 17.4 ± 4.8; *p* = 0.01) and personal accomplishment (21.8 ± 5.0 to 24.6 ± 4.3 vs. 21.9 ± 4.5 to 22.8 ± 4.3; *p* = 0.04). Significant correlations were found between change in emotional exhaustion and negative affect (positive correlation; r = 0.32; *p* = 0.034) and between personal accomplishment and perceived stress (negative correlation; r = −0.451; *p* = 0.002). This study demonstrates the potential of IBSR to improve teacher well-being. Future randomized studies are needed to evaluate the causality of IBSR in reducing burnout among teachers and other high-stress workplaces.

## 1. Introduction

Studies have demonstrated that one third of all new teachers experience high levels of burnout and ultimately leave their profession during the first three to five years [1]. This creates a shortage of teachers which leads to reductions in teaching quality and low educational standards [1,2].

The concept of *burnout* has been defined as a response to persistent emotional stress leading to reduced coping resources of an individual. According to [3] the three components of burnout are emotional exhaustion, reduced personal accomplishment and depersonalization. Emotional exhaustion is the main component and it represents a lack of mental resources due to emotional overload. Depersonalization refers to the feeling of alienation and negative attitude towards the surroundings. Decreased personal accomplishment refers to a person’s inability to produce desirable results due to a lack of external resources (e.g., feedback and evaluation) as well as internal resourcefulness (e.g., enthusiasm and interest) [4]. Burnout was found to be a chronic and progressive state, unlike temporal exhaustion, which passes after a short rest [3,4,5].

Teacher burnout can be explained by various personal and organizational characteristics [6]. The personal characteristics that predict teacher’s burnout include: (1) psychological factors, mainly unfulfilled expectations, a sense of meaninglessness and a lack of opportunities for personal accomplishment [7]. Other psychological factors include personal predispositions, such as sensitivity, ambitions, idealism, devotion and high work standards; (2) social factors such as difficult interactions with pupils and/or colleagues, lack of social support, lack of opportunities for cooperation and creativity and public criticism also significantly contribute to teacher’s burnout [4]. The organizational characteristics that predict teacher’s burnout include: (1) environmental conditions such as crowded classrooms and constant noise [8]; (2) factors related to the education system, such as bureaucracy and limitations on teachers’ autonomy [9]; (3) social factors such as decreased professional prestige and low income [10].

Organizational and individual tools have been developed to treat burnout [11]. The organizational tools aim to affect the social, institutional and environmental factors affecting teacher burnout. These tools include changes in classroom size, redefining job roles and offering teachers new instructional topics. These organizational tools are effective but less commonly implemented due to their large financial and organizational resource requirements [12]. Various *individual tools* also exist aimed at providing behavioral and psychological techniques for teachers to cope with work-related stress as well as empowering them by providing opportunities for occupational and personal development. These tools include time-management techniques, leadership and communication skills, strengthening self-image, Cognitive Behavioral Therapy (CBT), Positive Psychology Interventions (PPI) and mindfulness-based interventions such as mindfulness-based stress reduction (MBSR), transcendental meditation, prayer, breathing and yoga [13,14,15,16]. A Cochrane review in 2015 concluded that organizational interventions studied to date have had limited efficacy [17] and a systematic review of mindfulness across eight studies using MBSR as an intervention for job burnout yielded promising results [18], suggesting that individual tools may have utility for burnout, however the overall evidence of these studies was rated as poor to good quality (one poor, six fair and one good) and effect sizes were modest, indicating that there is an ongoing need for research on novel effective strategies for burnout and teacher burnout in particular.

Inquiry Based Stress Reduction (IBSR) is a cognitive reframing practice similar to Cognitive Behavioral Therapy that may have promise as an individual tool to prevent burnout, based on its previously reported efficacy in reducing depression, anxiety and stress [19,20,21,22,23].

IBSR, also called “The Work”, involves identifying and investigating stressful thoughts that cause stress and suffering [24]. IBSR shares the same fundamental assumption as the classical Cognitive Behavioral Therapy (CBT), namely that dysfunctional beliefs are the main cause of distress. However, the process of cognitive restructuring involved in IBSR is addressed by awareness and personal realization [25,26] rather than reasoning and argument as in CBT [27]. In addition, unlike CBT, IBSR is free and can be practiced alone or with others, and does not require a trained facilitator.

IBSR has been practiced by many individuals worldwide and its effectiveness has been demonstrated on various psychological scales including physical, social, emotional and functional wellbeing [1], depression, anxiety and hostility [20], stress, satisfaction and psychological well being [21], anxiety, depression [22], anger and quality of life [23]. However, IBSR’s potential efficacy for reducing burnout among teachers has not been tested to the best of our knowledge. The aim of the current study was to evaluate the effectiveness of IBSR in reducing burnout levels among teachers, a well-known phenomenon with significant personal and social implications.

## 2. Materials and Methods

### 2.1. Requirement and Study Procedure

This quasi-experimental study was carried out at Begin High School in Rosh Haayin, Israel. Teachers with previous experience with the IBSR technique were excluded from the study.

**Figure 1:**
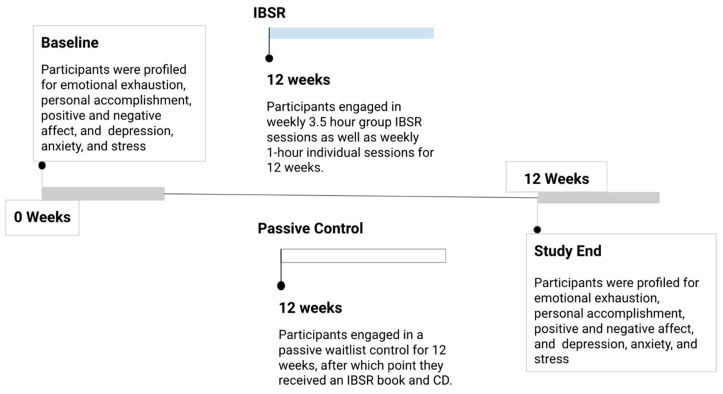
Study timeline. Participants were profiled at baseline for burnout and psychological well-being. They were assigned to Inquiry Based Stress Reduction (IBSR) or control and underwent either a 12-week IBSR training program or a 12-week waiting period. Psychological profiling and assessment of burnout was performed again at study week 12.

An advertisement for the study was published on the teachers’ billboard (at school and online). In addition, the study was announced during a teachers’ meeting. Teachers who wanted to participate met with a study coordinator and were informed about the study’s objectives and procedures. Teachers who agreed to participate signed an informed consent. The first twenty-five eligible teachers to enroll were included in the intervention group in order to ensure a full group. They completed a 12-week IBSR intervention. The rest of the teachers were included in the passive control group and received an IBSR kit (a book and a CD) at the end of the study, in order to reduce dropout rates (Figure 1). The study was carried out with the support of the school minister and was approved by both the Ethics Committee of Tel-Aviv University and the Israeli Ministry of Education (Approval-7310).

### 2.2. Data Collection

All participants completed the following questionnaires before and after the intervention (study baseline and study week 12). Baseline surveys were given 10 days before the intervention and all were collected before the intervention. Week 12 surveys were given at completion of intervention and were collected up to 10 days after.

1. Demographic questionnaire—includes demographic questions such as age, economic status, marital status, years of education, years of teaching, teaching role in school and degree of spirituality.
2. Maslach Burnout Inventory (MBI)—the most common questionnaire in the field of occupational burnout in the last 30 years [28,29]. Its validity and reliability were demonstrated in various studies [3,4] The MBI includes 14 items, which evaluate emotional exhaustion (8 items) and personal accomplishment (6 items) [30].
3. Positive and Negative Affect Scale (PANAS)—evaluates the emotional state of individuals and includes 10 items of positive affect and 10 items of negative affect. The questionnaire was found consistent on various time points; hence, it can be used as a state or trait scale [31].
4. Perceived Stress Scale (PSS)—evaluates the frequency in which a person perceives daily situations as stressful. It includes 14 items rated on a scale of 0 to 4. A higher score means a more stressful daily experience. This questionnaire validity was confirmed in previous studies [32].
5. Depression, Anxiety, Stress (DASS)—includes 21 items evaluating depression, anxiety and stress to identify a clinical state. This questionnaire was used in previous studies with teachers [33].

### 2.3. IBSR Intervention

The IBSR intervention included weekly group meetings (3.5 h/meeting) and weekly individual sessions with a facilitator (1 h/session) for 12 weeks. All the sessions were standardized according to a training manual and assessed to maintain consistency in the program. Participants were considered active if they were present in at least 75% of the group meetings and completed 50% of the home practice.

IBSR is a simple technique that involves three steps: Step (1) Participants identify stressful thoughts and write these stressful thoughts out on paper. The main tool for this task is the “Judge-Your-Neighbor” worksheet (Figure S1). Step (2) Guided reflection: participants, on their own or with the help of a “facilitator” (a person trained in the IBSR technique), select a thought they have written down and investigate each thought one at a time using a set of four guided questions: (1) Is it true? (2) Can I absolutely know that it is true? (3) How do I react when I believe that thought? (4) Who would I be without the thought? The self-investigation enables the participant to question their instinctive beliefs and examine their emotional and physical responses during stress evoking situations. This stage is meditative and the participants are encouraged to allow space to identify their own true answers to the four questions with no pre-defined agenda. The guidance is to be in a state of witnessing awareness, in which a person observes the thoughts that come into mind without trying to control or direct them [2]. The goal is realization, not rationalization. Step 3) Participants “turn around” their stressful thoughts. In the turnarounds, participants identify possible evidence for the opposite of the thought. For example, if the original thought was: “My students don’t listen to me,” possible turnarounds can be: “I don’t listen to my students” (turnaround to the other), “I don’t listen to myself” (turnaround to the self) and “My students do listen to me” (turnaround to the opposite). The participants are asked to find three genuine examples in which the turnaround is as true as the original thought. By doing so, the participants may learn that there are other possible interpretations and learn to interpret the world through a less stressful lens. This way, situations perceived as stressful can now be experienced with peace of mind and connectedness [27].

### 2.4. Statistical Analysis

In accordance with previous studies, which examined mental burnout as the main outcome of similar interventions for teachers [16,18], we used “emotional exhaustion” as an anchor with an expected difference of 3.5 units and a standard deviation of 4.5 units. Based on a 5% alpha and a power of 80%, we found that a sample size of 54 participants was sufficient to demonstrate a statistically significant difference between the groups. SPSS 19 software was used for statistical analyses. In order to assess selection bias, we assessed group differences at baseline using independent *t*-tests between groups. Pearson correlation coefficients were used to examine the correlation between the dependent variables and a two-way analysis of variance (ANOVA) with repeated measures was used to examine interactions between group and time factors. To assess dropout bias, a comparison was carried out between participants who completed the intervention and those who dropped out.

## 3. Results

### Study Cohort and Baseline Demographics

One hundred and forty high school teachers were found eligible to participate in the study. Sixty were enrolled and seven teachers dropped out before filling out the baseline questionnaires or completing the intervention. They were not included in the analysis. For the teachers that filled out baseline questionnaires and began the intervention (*n* = 53), Table 1 details the demographic characteristics of the control (*n* = 28) and intervention groups (*n* = 25). No significant differences in the study’s scales were found between the two groups before the intervention (Table S1). In addition, seven individuals dropped out during the course of the intervention. No significant differences in baseline metrics were identified between dropouts and completers (Table S2). Additionally, Cronbach’s alpha was high (0.79–0.91) across all scales, indicating high internal consistency (Table S3).

**Figure 2:**
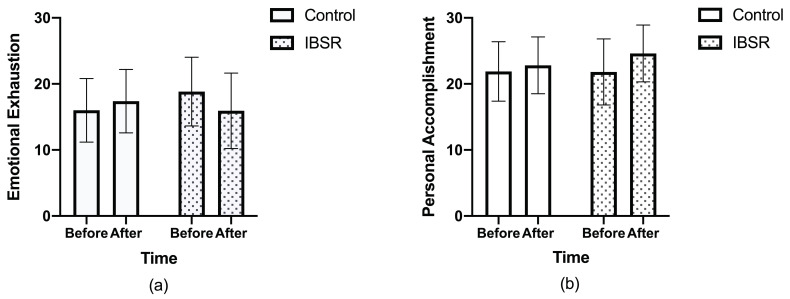
Change in burnout parameters. (**a**) Change in emotional exhaustion over the course of the study for the IBSR intervention group and control; (**b**) change in personal accomplishment over the course of the study for the IBSR intervention group and control. Data are displayed as means +/− standard deviation.

Relative to the control group, teachers in the intervention group showed greater improvement in emotional exhaustion (18.8 ± 5.2 to 15.9 ± 5.7), compared with the control group (16.0 ± 4.8 to 17.4 ± 4.8) (Table 2, Figure 2a). The difference between the groups was found statistically significant (*p* = 0.01). Personal accomplishment was increased in the intervention group (21.8 ± 5.0 to 24.6 ± 4.3), as well as in the control group (21.9 ± 4.5 to 22.8 ± 4.3). The difference between the groups was found to be statistically significant (*p* = 0.04) (Figure 2b). A decrease was found in the perceived stress in the intervention group (27.7 ± 5.5 to 22.4 ± 5.8), which was greater than the control group (24.6 ± 9.3 to 22.7 ± 10.0). However, this difference was not statistically significant (*p* = 0.1) (Table 2). Teachers in both groups did not have clinical distress at baseline.

Significant changes in DASS scales were not observed within or between groups (Table 2). The correlations matrix (Table 3) reveals the following findings: significant positive correlations were found between change in emotional exhaustion and negative affect (r = 0.32, *p* = 0.03). A significant negative correlation was found between personal accomplishment and perceived stress (r = −0.45, *p* < 0.01).

## 4. Discussion

The results of the current study demonstrate that burnout parameters of emotional exhaustion and personal accomplishment improved significantly in the intervention group, compared with the control group, suggesting that IBSR may be a viable approach to reducing teacher burnout (Figure 2). Relative to the control group, teachers in the intervention group showed greater improvements in emotional exhaustion (18.8 ± 5.2 to 15.9 ± 5.7 vs. 16.0 ± 4.8 to 17.4 ± 4.8, *p* = 0.01) and personal accomplishment (21.8 ± 5.0 to 24.6 ± 4.3 vs. 21.9 ± 4.5 to 22.8 ± 4.3, *p* = 0.04). These results may also have clinical significance, given known correlations between burnout and hypertension, hyperlipidemia, cardiovascular diseases and mortality [34,35,36]. Further studies are warranted to validate the effect of IBSR on teacher burnout and explore whether IBSR-mediated improvements in teacher burnout are associated with improved psychological and physiological outcomes in the long-term.

To gain a deeper understanding of how IBSR may mediate improvements in teacher burnout, the present study assessed improvements in mental health and correlations between burnout indicators and other psychometrics, including positive and negative affect. A negative correlation was found between personal accomplishment and perceived stress (r = −0.451, *p* = 0.002). In addition, a positive correlation was identified between emotional exhaustion and negative affect (r = 0.32, *p* = 0.034). These findings are consistent with previous studies in the field of occupational burnout, which demonstrated that these psychological scales correlated with burnout parameters [37,38]. Perceived stress improved over time across groups (time factor *p* = 0.002). The present study tended to have greater improvements in perceived stress in the IBSR group as compared to control (*p* = 0.1). Further studies with greater power are needed to resolve whether improvements in perceived stress and positive and negative affect may contribute to improvements in personal accomplishment and emotional exhaustion or vice versa.

In addition to effectiveness, the 90% adherence rate to the program observed in the present study suggests that IBSR may be a well-tolerated and well-suited intervention for teacher burnout. Previous literature suggests that burnout interventions should relate to teachers’ personal beliefs regarding sources of stress and their physiological effects [13,39]. The format of IBSR fits this objective, because it focuses on an inquiry process, which enables people to investigate their attitudes regarding various aspects of teaching, for example discipline and responsibility over pupils, which are highly correlated to burnout [40,41].

We are aware of several limitations in the current study. First, the assignment was not randomized; however, it should be noted that a comparison between the intervention and the control group did not reveal any difference between demographic characteristics and psychological scales on baseline. Second, the average number of years in teaching was 18, with only two teachers in the intervention group working less than five years in teaching. Hence, it cannot be concluded whether IBSR is effective for increasing retention of new teachers [42]. In addition, the study did not include a longitudinal follow-up period. Hence, the long-term effects of the intervention cannot be evaluated and given the promising improvements seen in burnout parameters in the IBSR group, further studies are warranted to evaluate prolonged effects. Finally, in this study we have not explored how the intervention reduces teachers’ burnout. Further studies are warranted to investigate the mechanisms underlying the effects of IBSR on mental health.

## 5. Conclusions

The IBSR intervention was associated with improvement in emotional exhaustion and personal accomplishment, demonstrating the potential efficacy of IBSR for reducing teacher burnout. Given its low costs and minimal organizational involvement, IBSR could be implemented as a tool for promoting teachers’ well-being. Future randomized controlled studies could evaluate the effectiveness of IBSR on alleviating stress and burnout and enhancing well-being levels in the workplace, particularly among new teachers and those with higher depression, anxiety and stress. Overall, this study demonstrates the promise of inquiry-based approaches for promoting well-being and reducing burnout among teachers.

## Figures and Tables

**Table 1:** Baseline demographics.

Characteristic		Intervention (*n* = 25) Mean (SD)	Control (*n* = 28) Mean (SD)	*p* Value
Age		46.3 (6.5)	46.5 (6.1)	t (52) = 0.12, *p* = 0.90
Level of Education (years)		17.5 (2.3)	17.7 (2.2)	t (49) = 0.27, *p* = 0.79
Teaching Experience (years)		18.2 (8.1)	18.2 (8.8)	t (52) = 0.26, *p* = 0.98
Occupation (percent time working)		114.8 (24.6)	109.4 (31.6)	t (52) = −0.67, *p* = 0.49
Sex N (%)	Male	1 (3.8%)	6 (21.4%)	χ^2^ = 3.69, *p* = 0.12
	Female	24 (96.2%)	22 (78.6%)	
Marital Status N (%)	Married	(92%) 23	19 (67.9%)	χ^2^ = 4.68, *p* = 0.32
	Single	(8%) 2	9 (32.1%)	
Economic status N (%)	Above average	37.5%	46.2%	χ^2^ = 0.38, *p* = 0.37
	Below average	62.5%	53.8%	

SD = Standard deviation, occupation is amount of time working, 100% = full time.

**Table 2:** Teacher burnout and well-being before and after the intervention.

Study Outcome	Intervention Mean (SD)		Control Mean (SD)		Between-Group
	Before	After	Before	After	Difference (ANOVA)
Burnout—Emotional Exhaustion	18.8 (5.2)	15.9 (5.7)	16.0 (4.8)	17.4 (4.8)	F(1,45) = 13.2, *p* = 0.01 *
Burnout—Personal Accomplishment	21.8 (5.0)	24.6 (4.3)	21.9 (4.5)	22.8 (4.3)	F(1,44) = 4.7, *p* = 0.04 *
Perceived Stress Scale (PSS)	27.7 (5.5)	22.4 (5.8)	24.6 (9.3)	22.7 (10.0)	F(1,42) = 2.59, *p* = 0.12
PANAS—Positive Affect	39.4 (6.2)	40.7 (5.1)	39.7 (7.6)	40.8 (6.3)	F(1,42) = 0.10, *p* = 0.92
PANAS—Negative Affect	42.2 (7.6)	43.9 (5.0)	42.9 (6.7)	43.8 (5.6)	F(1,42) = 0.23, *p* = 0.63
DASS—Depression	2.6 (2.8)	2.3 (4.4)	2.5 (4.2)	1.7 (2.0)	F(1,43) = 0.44, *p* = 0.51
DASS—Anxiety	1.8 (1.8)	1.7 (4.44)	1.8 (3.4)	1.0 (1.7)	F(1,43) = 0.03, *p* = 0.87
DASS—Stress	6.6 (4.4)	5.8 (4.5)	5.4 (4.9)	4.7 (3.5)	F(1,43) = 0.44, *p* = 0.51

SD = Standard deviation, * *p* value < 0.05.

**Table 3:** Correlation matrix between study outcomes.

Measure	Emotional Exhaustion	Personal Accomp.	Perceived Stress	Pos. Affect	Neg. Affect	Stress	Anxiety	Depression
Emotional Exhaustion	1	−0.28	0.27	−0.14	0.32 *	−0.07	−0.13	−0.21
Personal Accomp.	−0.28	1	−0.45 *	0.048	−0.25	−0.08	0.28	0.18

* Values marked in bold are statistically significant at a cutoff of α = 0.05. Pos., Positive; Neg, Negative; Accomp, Accomplishment.

## Supplementary Materials

The following are available online at https://www.mdpi.com/2076-3425/10/7/468/s1, Table S1: Comparison of psychological scales at baseline between the intervention and control groups, Table S2: Dropouts vs. completers, Table S3: Cronbach’s alpha for psychological scales, Figure S1: Judge-Your-Neighbor Worksheet.
