# Supplementary material for: The Effect of Inquiry-Based Stress Reduction on Teacher Burnout: A Controlled Trial
Source: Brain Sci. 2020 Jul 21;10(7):468. doi: 10.3390/brainsci10070468 (PMC7407507; doi:10.3390/brainsci10070468)
Supplement: Supplementary file 1 [file brainsci-10-00468-s001.pdf]

Supplementary Materials

Table S1. Comparison of psychological scales at baseline between the intervention and control groups.

Variables	Internal Mean	Control Group Average (SD)	Intervention Group Average (SD)	T Test for Objective Samples
Mental Burnout	*n*	28	25	
	Emotional Exhaustion	16.53 (4.89)	18.32 (5.32)	t = −1.273 df = 51 *p* = 0.209
	Personal Accomplishment	22.57 (4.67)	22.84 (4.66)	t = 0.569 df = 51 p = 0.572
Perceived Stress Scale (PSS)	n	27	24	t = −1.611 df = 49 p = 0.113
	(no internal division)	24.59 (8.47)	27.83 (5.31)	
Positive and Negative Affect Scale	*n*	27	25	
	Positive affect	39.29 (7.57)	39.29 (6.09)	t = 0.002 df = 49 *p* = 0.998
	Negative affect	43.14 (6.25)	42.04 (7.54)	t = 0.572 df = 49 *p* = 0.570
Depression, Anxiety, Stress (DASS)	*n*	28	21	
	Stress	5.22 (4.56)	7.00 (4.08)	t = −1.459 df = 49 *p* = 0.151
	Anxiety	1.59 (3.12)	2.12 (2.07)	t = −0.707 df = 49 *p* = 0. 483
	Depression	2.22 (3.94)	2.87 (2.81)	t =−0.0672 df = 49 *p* = 0.505

Table S2. Dropouts vs. completers.

Measurement		Completers Average (SD)	Dropout Group Average (SD)	T test for Independent Samples (SD)
Mental Burnout	*n*	45	8	
	Emotional exhaustion	17.41 (5.19)	16.87 (4.70)	t = 0.498 df = 52 *p* = 0.785
	Personal accomplishment	21.84 (4.69)	25.25 (3.65)	t = .0624 df = 52 *p* = 0.057
PSS	*n*	44	8	
		26.00 (7.80)	26.75 (1.98)	t = −0.286 df = 50 *p* = 0.790
PANAS	*n*	44	8	

	Positive affect	39.59<br>(6.79)	37.00<br>(7.03)	t = .0987<br>df = 50<br>p = 0.328
	Negative affect	42.45<br>(7.05)	44.00<br>(5.34)	t = −0.588<br>df = 50<br>p = 0.559
DASS	n	44	8	
	Stress	6.09<br>(4.62)	5.25<br>(3.01)	t = 0.493<br>df = 50<br>p = 0.623
	Anxiety	1.84<br>(2.68)	1.62<br>(2.66)	t = .0209<br>df = 50<br>p = 0.835
	Depression	2.63<br>(3.57)	1.62<br>(2.44)	t = .0765<br>df = 50<br>p = 0.84

Table S3. Cronbach's alpha for psychological scales.

**Cronbach's Alpha After**	**Cronbach's Alpha Before**		
0.788	0.793	Emotional exhaustion	**MBI—**
0.798	0.786	Personal accomplishment	
0.831	0.887	Stress	**DASS—**
0.898	0.738	Anxiety	
0.912	0.891	Depression	
0.852	0.871	Positive affect	**PANAS**
0.880	0.910	Negative affect	
0.894	0.877		**PSS**

THE WORK of Byron Katie®

Judge-Your-Neighbor Worksheet

Judge your neighbor • Write it down • Ask four questions • Turn it around

Think of a recurring stressful situation, a situation that is reliably stressful even though it may have happened only once and recurs only in your mind. Before answering each of the questions below, allow yourself to mentally revisit the time and place of the stressful occurrence.

1. In this situation, time, and location, who angers, confuses, or disappoints you, and why?

I am ____________ with ______________ because ______________________________________

emotion *name*

____

Example: I am *angry* with *Paul* because *he doesn't listen to me about his health.*

2. In this situation, how do you want them to change? What do you want them to do?

I want ______________ to ____

name

____

____

Example: I want *Paul* to *see that he is wrong. I want him to stop smoking. I want him to stop lying about what he is doing to his health. I want him to see that he is killing himself.*

3. In this situation, what advice would you offer to them?

________________ should/shouldn't ______________________________________

name

____

____

____

Example: Paul should *take a deep breath. He should calm down. He should see that his actions scare me and the children. He should know that being right is not worth another heart attack.*

4. In order for *you* to be happy in this situation, what do you need them to think, say, feel, or do?

I need ________________ to ____

name

____

____

Example: I need *Paul* to *hear me. I need him to take responsibility for his health. I need him to respect my opinions.*

5. What do you think of them in this situation? Make a list.

__________________ is ____

name

____

Example: Paul is *unfair, arrogant, loud, dishonest, way out of line, and unconscious.*

6. What is it in or about this situation that you don't ever want to experience again?

I don't ever want ____

____

Example: I don't ever want *Paul to lie to me again. I don't ever want to see him smoking and ruining his health again.*

The four questions

Example: Paul doesn't listen to me about his health.

1. **Is it true?** (Yes or no. If no, move to 3.)
2. **Can you absolutely know that it's true?** (Yes or no.)
3. **How do you react, what happens, when you believe that thought?**
4. **Who would you be without the thought?**

Turn the thought around

a) **to the self.** (*I* don't listen to myself about *my* health.)
b) **to the other.** (*I* don't listen to *Paul* about his health.)
c) **to the opposite.** (Paul *does* listen to me about his health.)

Then find at least three specific, genuine examples of how each turnaround is true for you in this situation.

The turnaround for statement 6:

I am willing to ______________________________ *(Example: I am willing to have Paul lie to me again.)*

I look forward to ____________________________ *(Example: I look forward to having Paul lie to me again.)*

For more information on how to do The Work, visit thework.com

© 2013 Byron Katie International, Inc. All rights reserved. thework.com Rev. 8 Jan 2013

Figure 1. Judge−Your−Neighbor Worksheet.

*Permission has been obtained for use of copyrighted material
